# Manganese Oxide Nanochips as a Novel Electrocatalyst for Direct Redox Sensing of Hexavalent Chromium

**DOI:** 10.1038/s41598-019-44525-4

**Published:** 2019-05-29

**Authors:** Gaurav Bhanjana, Pooja Rana, Ganga Ram Chaudhary, Neeraj Dilbaghi, Ki-Hyun Kim, Sandeep Kumar

**Affiliations:** 1 Department of Chemistry & Centre of Advanced Studies in Chemistry, Panjab University, 160014 Chandigarh, India; 2 Department of Bio and Nano Technology, Guru Jambheshwar University of Science and Technology, 125001 Hisar, Haryana India; 3 Department of Civil & Environmental Engineering, Hanyang University, 222 Wangsimni-Ro, 04763 Seoul, Republic of Korea

**Keywords:** Sensors and biosensors, Nanosensors

## Abstract

In order to maintain a healthy organisation of bionetworks, both qualitative and quantitative estimation of hexavalent chromium in food and beverage samples is required based on proper quality control and assurance. Nonetheless, conventional quantitation techniques for hexavalent chromium generally suffer from certain limitations (e.g., the need for expertise, costly equipment, and a complicated procedure). This research was performed to elaborate a novel method to quantify hexavalent chromium based on an electrochemical cyclic voltammetry technique. To this end, nanochips of manganese oxide (Mn_3_O_4_: approximately 80–90 nm diameter and 10 nm thickness) were synthesized using a chemical method and characterized with spectroscopic and microscopic approaches. These nanochips were employed as proficient electrocatalytic materials in direct redox sensing of hexavalent chromium in both real samples and laboratory samples. Manganese oxide nanochips felicitated large surface area and catalytic action for direct electrochemical reduction of hexavalent chromium at electrode surface. This fabricated nanochip sensor presented a detection limit of 9.5 ppb with a linear range of 50–400 ppb (sensitivity of 25.88 µA cm^−2^ ppb^−1^).

## Introduction

Endocrine-disrupting compounds (EDCs) are substances present in our food, water, environment, and other consumer-based products that interfere with hormones or any system controlled by hormones^1^. Dioxins, biphenyls, heavy metals, plasticizers, detergents, perfluorinated chemicals, cosmetics, and pesticides are some major sources of endocrine disruptors. EDCs are disease causative agents in brain development and many other well-known diseases in human body (e.g., cancer, deformations, and certain neural disorders). EDCs are also responsible for reduced fertility, feminizing of males, and masculinizing character of females as they hinder the reproductive system and/or sexual development. EDCs act by interfering with signals, binding, transport, elimination, synthesis, and secretion of natural hormones.

Among various EDCs, heavy metals represent one of the most common substances present in our ecosystem. Most of the metals observed in the environment are due both to geologic and anthropogenic origins^2,3^. Among different heavy metals, the presence of hexavalent chromium in the bionetwork is identified ubiquitously from plants, animals, rocks, and volcanic eruptions. Chromium (VI) has extensive applications in the leather industry, wood industry, electroplating, and stainless-steel production. Hexavalent chromium enters the human body through breathing and/or the skin or after being mixed with food and water. It is transported directly into the cells through sulphate channels and causes genotoxicity^4,5^. After being reduced to chromium (III), it forms stable compounds with proteins and nucleic acid that are responsible for mutagenic damage. Haemolysis, kidney failure, liver failure, dermatitis, and other allergic reactions are also consequences of chromium toxicity.

Direct exposure to chromium can be reduced or avoided by proper and routine monitoring of samples used for human consumption. Hence, there is a requirement of qualitative and quantitative estimation of hexavalent chromium in food, beverages, and many other environmental media. The Environmental Protection Agency has set the maximum admissible limit of total chromium in drinking water as 100 ppb^6,7^. Atomic absorption spectroscopy (AAS), ICP-MS (inductively-coupled plasma-mass spectrometry), X-ray fluorescence spectrometry, optical emission spectroscopy (OES), and WD-XRF (wavelength dispersive X-ray fluorescence spectrometry) are techniques that are presently employed for the estimation of hexavalent chromium^8–10^. Although these techniques are quite efficient, they suffer from certain limitations like requiring expertise, a large sample volume, recurring cost, and a complex pre-treatment procedure^10–14^. Another important technique which is used widely for detection of hexavalent chromium is stripping voltammetry. In this technique, the target metal is deposited onto the working electrode. Although the technique is suitable for the detection of chromium ions but the process used for fabrication of electrode in this process is quite convoluted. Apart from this, toxicity of mercury electrode used during analysis of heavy metals is major concern in stripping voltammetry. The inability to differentiate closely related chemical species is crucial shortcoming associated with stripping voltammetry technique. Additionally, complex procedure, pre-concentration steps, and requirement of skilled person to operate this instrument limit its applications further^13–16^.

The potential of electrochemical methods for heavy metal quantitation has drawn great attention due to numerous merits (e.g., sensitivity, selectivity, easy handling, small sample volume, reliability, short response time, and portable on-site detection)^14–17^. Particularly, the specific electrode potential value of hexavalent chromium makes it an appropriate contender for direct redox sensing^17^. Nanomaterial-modified electrodes have offered a breakthrough in achieving high sensitivity, linearity, enhanced catalytic activity, selectivity, and other vital parameters in electrochemical sensing^18–20^. Nanomaterials have revolutionized the field of science and technology including sensing, catalysis, diagnosis, drug delivery, medicines, and packaging. In the case of sensing applications, nanomaterials like carbon-based and metal/metal oxide nanoparticles have set a milestone due to their superior properties (e.g., high surface area to volume ratio, chemical inertness, ease of synthesis, tuneable band gap, high electron communication features, quantum effects, biocompatibility, and ease of chemical modification)^19–23^.

Out of various metal oxide nanomaterials, manganese oxide nanoparticles hold an inimitable position with many distinguishable assets like high electrochemical stability, low cost, high catalytic activity, ease of synthesis, accessibility in various shapes, and ability to deliver high charge in little time. Manganese oxide is found to be an efficient catalyst in redox conversion of various compounds like methane, carbon monoxide, nitrobenzene and other organic compounds^18,20–24^. Accordingly, manganese oxide nanoparticles are well characterized with respect to their elemental, structural, optical, and compositional behaviour. As these categorized nanoparticles were evaluated as an efficient electro-catalyst, the electrochemical technique became a competent probe for quantitation of hexavalent chromium. Cyclic voltammetry (CV) was used as effective tools for its direct determination by considering the effects of solution pH, scan rate, and analyte concentration. This is the first report on the direct electrochemical sensing of hexavalent chromium using manganese oxide nanoparticles based on a CV technique. The use of this fabricated sensor allowed us to realize enhanced detectability (high sensitivity) and selectivity against Cr(VI). This research article provides a novel method for facile and direct estimation of hexavalent chromium in real and laboratory samples.

## Results and Discussion

**Figure 1:**
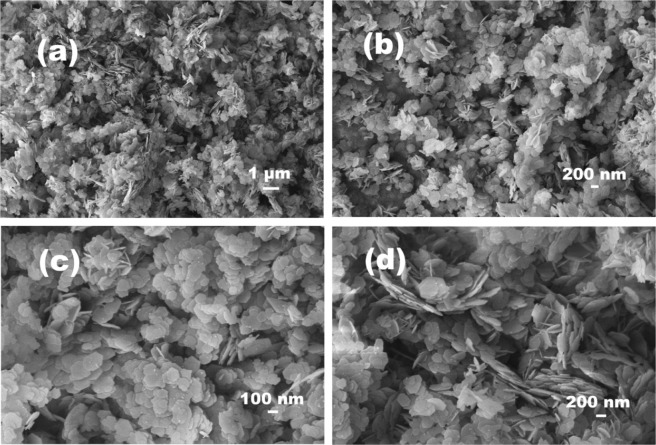
The morphological features of synthesized Mn_3_O_4_ nanochips acquired using FESEM (**a**) low magnification and (**b**,**c**,**d**) high magnification.

Refluxing was used as a chemical tool for the synthesis of manganese oxide nanoparticles. The size and morphology of synthesized nanoparticles were confirmed with electron microscopy. The exact size, morphology, and topology of the nanoparticles were also assessed with field emission scanning electron microscopy (FESEM). FESEM micrographs at various resolutions from different angles are shown in Fig. 1. As shown in the FESEM image, the synthesized nanoparticles were obtained at high yield. Moreover, the obtained nanochips had consistent symmetric features, with dimensions of 80–90 nm (diameter) and 10 nm (thickness). Images at higher and lower magnifications are provided in Fig. 1(a–d).

**Figure 2:**
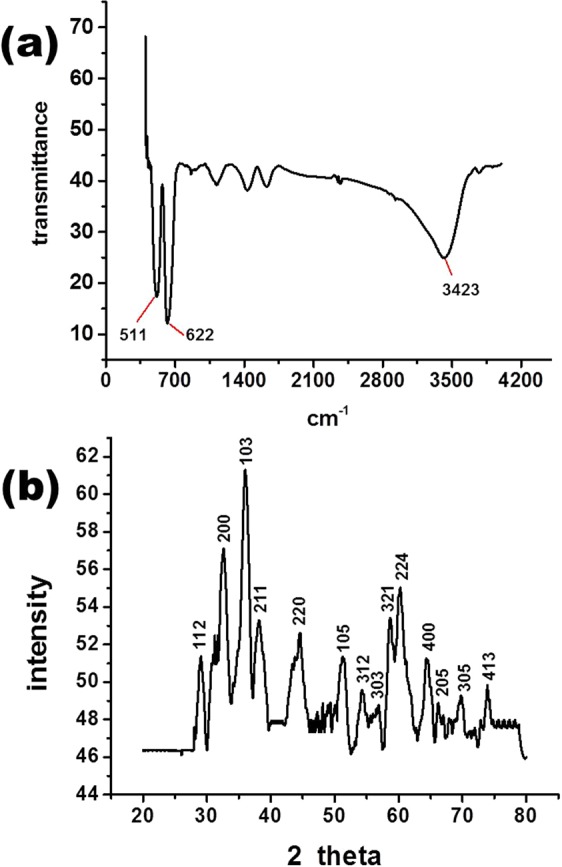
Spectra of synthesized Mn_3_O_4_ nanochips: (**a**) FTIR and (**b**) XRD.

FTIR spectroscopy was used as an efficient tool to assess the elemental nature of synthesized nanomaterial. As shown in the FTIR spectrum (Fig. 2a), the synthesized nanomaterial was pure manganese oxide without impurities. In the FTIR spectrum, peaks at 511 cm^−1^ and 622 cm^−1^ can be attributed to the presence of vibrations of the Mn-O bond, while the broad peak at 3423 cm^−1^ was due to stretching vibrations of the H-bonded surface water molecules and hydroxyl groups^25,26^. The XRD spectrum was used to explore the crystal structure of the synthesized nanomaterial. As illustrated in Fig. 2b, the synthesized nanomaterial had all characteristic reflections of pure manganese oxide (Mn_3_O_4_). Furthermore, the synthesized material was confirmed as Mn_3_O_4_ with a tetragonal structure (JCPDS Card 01-080-0382)^25,26^.

**Figure 3:**
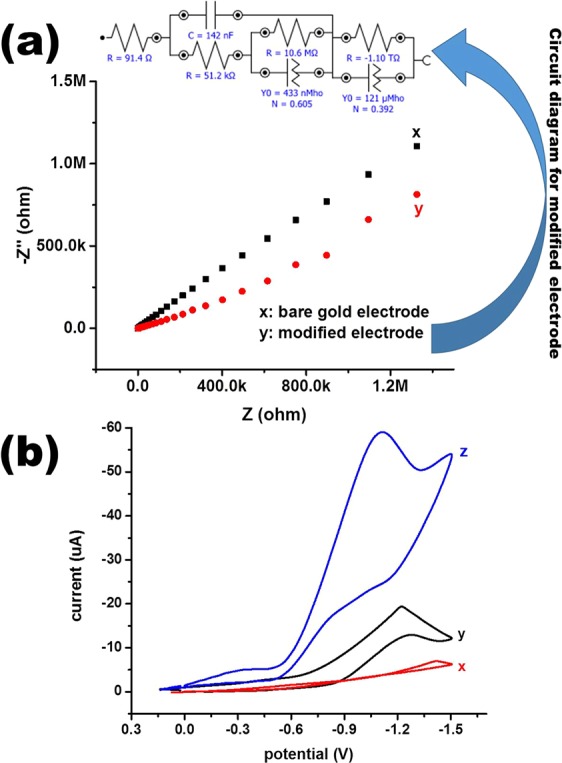
(**a**) Impedance plots for bare Au (x) and fabricated Mn_3_O_4_/Nafion/Au (y) electrodes with a circuit diagram of the fabricated electrode, (**b**) CV plots in the presence of a 150 ppb aqueous Cr(VI) solution with reference to: (x) blank, (y) using bare Au, and (z) fabricated Mn_3_O_4_/Nafion/Au electrodes (pH 2, scan rate 100 mV/s).

The results of these analyses clearly confirmed that the synthesized material was manganese oxide nanomaterial without impurities. This well characterized nanomaterial was exploited as efficient electro-catalyst for direct redox sensing of hexavalent chromium. The catalytic behaviour of synthesized manganese oxide nanochips was elucidated with the assistance of impedance spectroscopy. The impedance plot of the bare gold electrode and fabricated Mn_3_O_4_/Nafion/Au electrode is depicted in Fig. 3a.

Based on the data obtained in Fig. 3, the fabricated electrode has high electron communication in terms of conductivity. This change in behaviour is due to the presence of manganese oxide nanochips on the electrode surface, which imitate them as efficient electrocatalytic materials for electrode modification. This upshot in the presence of the Mn_3_O_4_ nanochips was due to the formation of the extended ordered defect structure caused by inflection at the atomic scale. This also offers favourably exposed sites with superior electrocatalytic activity and enhanced electronic structure. This phenomenon boosted the charge propagation at the electrode/electrolyte interface due to the development of an electrical double layer resulting from the minimum contact resistance at the interface^27–29^. A circuit diagram for the fabricated Mn_3_O_4_/Nafion/Au electrode is also portrayed in Fig. 3a. It can be inferred from Fig. 3b that a significant response in the reduction peak was obtained in the presence of hexavalent chromium using a fabricated Mn_3_O_4_/Nafion/Au electrode^30,31^.

The proposed reaction for the observed change is given in Eq. 1. On the basis of the calculations, the total number of electrons involved in the reduction process was 3.

1 $$HCr{O}_{{\rm{4}}}^{-}+7{H}^{+}+3{e}^{-}\to C{r}^{3+}+4{H}_{2}O$$

**Figure 4:**
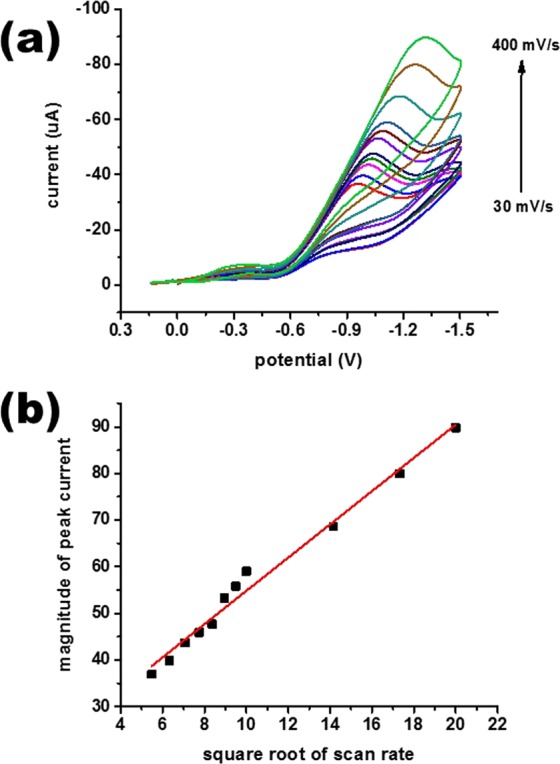
Basic performance of the fabricated Mn_3_O_4_/Nafion/Au electrode against 150 ppb aqueous Cr(VI): (**a**) CV plots in pH 2 solution at varying scan rates (30 mV/s-400 mV/s) and (**b**) plot of peak current vs. square root of scan rate.

There was also the appearance of a signal with low intensity in the presence of a bare Au electrode. In contrast, a difference was observed in the current obtained using the fabricated electrode for the same solution (concentration) of chromium ions. It clearly demonstrates that the synthesized manganese oxide nanoparticles were acting as a competent catalytic material in the redox transformation of hexavalent chromium. Another vital point was also observed in Fig. 3b in that the electrochemical signal was irreversible with a reduction peak only. There was no oxidation peak achieved during the reverse cycle, which clearly reflects that the electrochemical signal is irretrievable in used potential range. The electrocatalytic activity of the fabricated Mn_3_O_4_/Nafion/Au electrode was also established from the plot (Fig. 3b), and there was no redox peak in the absence of chromium. The effect of scan rate was also considered to elucidate the nature of the redox process, as demonstrated in Fig. 4.

**Figure 5:**
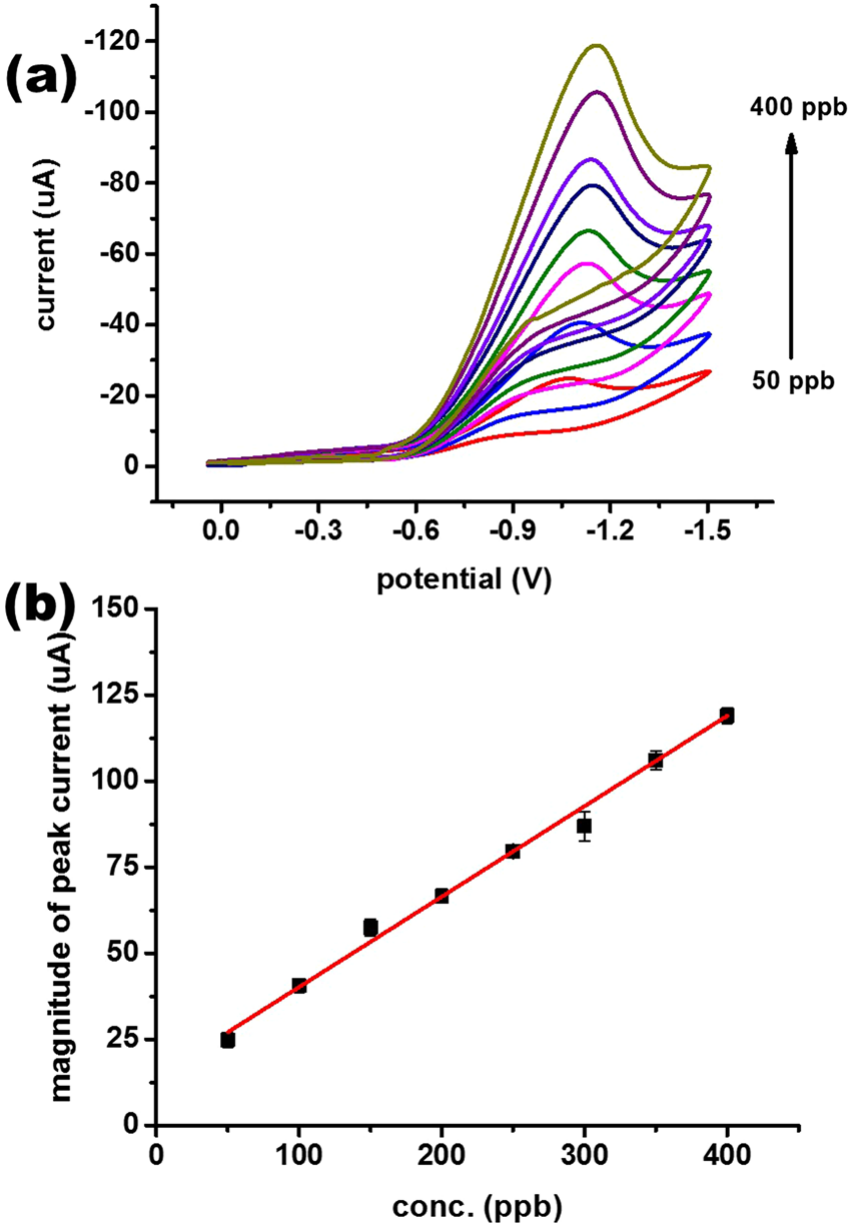
Basic performance of a fabricated Mn_3_O_4_/Nafion/Au electrode against 50–400 ppb of aqueous Cr(VI): (**a**) CV plots in the presence of varying concentrations at pH 2 at a scan rate of 100 mV/s and (**b**) plot of peak current vs. concentration.

Figure 4a validates that there is a regular increase in peak current with an increase in scan rate value. The linearity range of the fabricated electrode used in the present sensing system was examined by plotting the relationship between peak current and square root of scan rate, as depicted in Fig. 4b. There is a shift in the peak current with an increase in scan rate that clearly demonstrates that the process is diffusion controlled. The number of electrons involved in the reduction process were determined using Randles-Sevick equation and found to be 3. The number of electrons involved in the process illustrates that the hexavalent chromium is changing its oxidation state from +6 to +3. This conversion can also be validated from the fact that the obtained peak in CV is a reduction peak that also confirms that the obtained peak is only due to the reduction process. The effect of chromium concentration on peak current was also standardized as plotted in Fig. 5.

**Figure 6:**
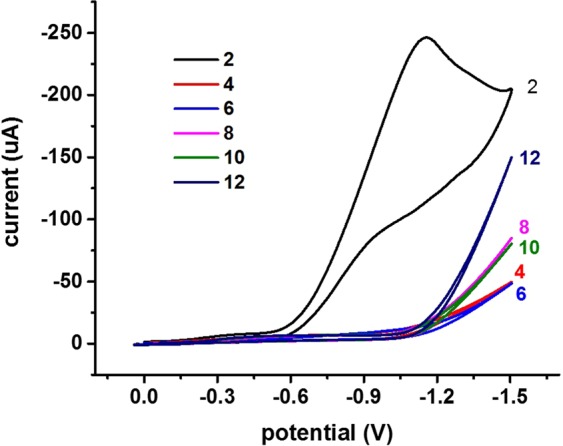
CV response of the fabricated Mn_3_O_4_/Nafion/Au electrode across different pH values at a scan rate of 100 mV/s.

Changes in concentration of chromium (VI) were assessed in relation to the corresponding current using the CV technique with a fabricated sensor and proposed technique. In Fig. 5a, there is a gradual increase in peak current with an increase in chromium (VI) concentration. Linearity between peak current and concentration is reflected in Fig. 5b. The detection limit of the proposed sensor, when calculated in terms of the limit of detection (LOD) using the formula (3*SD/calibration slope), was 9.5 ppb. The sensitivity of the fabricated sensor was also calculated and determined to be 25.88 µA cm^−2^ ppb^−1^. Based on the preliminary experiments, solution pH was also a fundamental factor for redox sensing of hexavalent chromium. The effect of solution pH on electrochemical response is illustrated in Fig. 6. The fabricated Mn_3_O_4_/Nafion/Au electrode exhibited a significant electrochemical response at only a pH of 2. There was no appearance of any peaks with the fabricated sensor at any other pH value. This inclination can be easily explained by the fact that there is a necessity of H^+^ ions for reduction of hexavalent chromium (as is clear from Equation 3). Alternatively, as the solution became more basic, there is no availability of a large number of H^+^ ions. Hence, the reduction of hexavalent chromium cannot be achieved at basic pH values.

**Figure 7:**
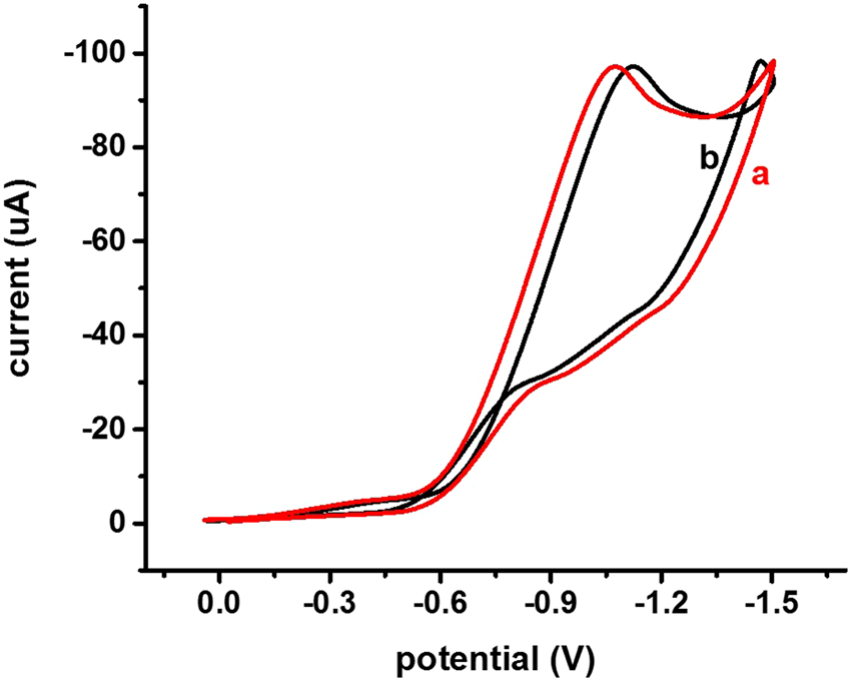
CV plots of the fabricated Mn_3_O_4_/Nafion/Au electrode in aqueous solution containing Cr(VI) in the (**a**) absence and (**b**) presence of other interfering ions.

The selectivity of the fabricated Mn_3_O_4_/Nafion/Au electrode towards chromium was also measured as presented in Fig. 7. CV plots with the fabricated electrode, when recorded in the presence and absence of possible interfering ions (other than hexavalent chromium), are depicted in Fig. 7. All possible interfering ions were taken simultaneously so as to provide the same environment as equivalent to real samples. In addition to As^3+^ and Hg^2+^ ions, some other cations (e.g., Pb^2+^, Zn^2+^, Cu^2+^, Cd^2+^, and Mn^2+^) were also considered for potential interference. These specific interfering ions were considered in the present work because these are the potentially existing interfering agents/ions with chromium in water samples. Moreover, the chances of interference from these ions are more in considered potential range for chromium detection. An identical peak current was attained in both the presence and absence of these ions.

**Figure 8:**
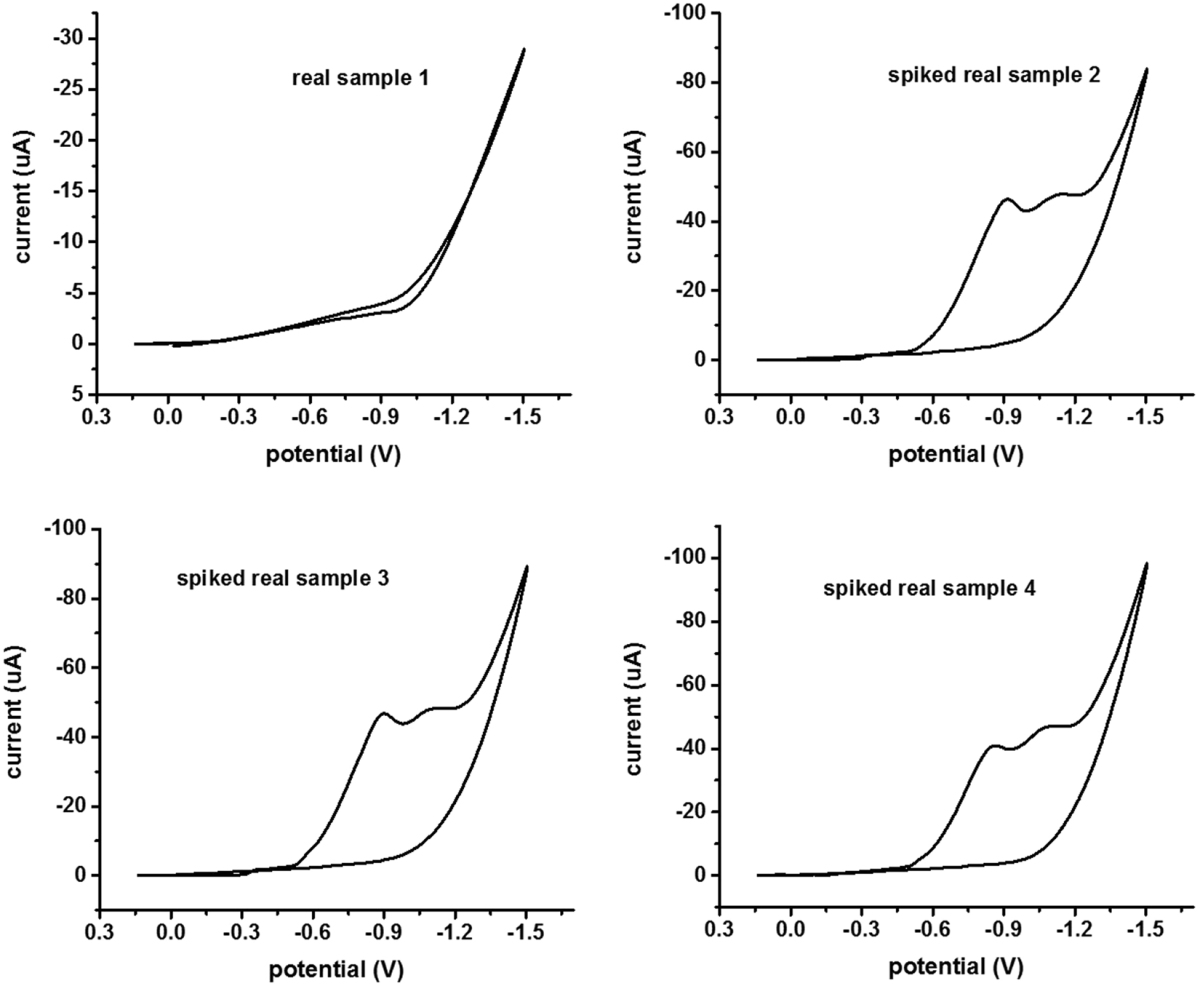
CV plots for real samples using a fabricated Mn_3_O_4_/Nafion/Au electrode (scan rate 100 mV/s, pH value 2).

As shown in Fig. 7, a reproducible signal was obtained in the CV cycle even in the presence of other interfering ions. This outcome clearly indicates that the proposed CV technique and fabricated Mn_3_O_4_/Nafion/Au electrode were selective towards hexavalent chromium. The reason behind selectivity is the specific electrode potential of every metal ion which prevent other metal ions to produce signal at same potential value. Real environmental samples were also analysed using the fabricated Mn_3_O_4_/Nafion/Au electrode with CV technique. CV plots obtained for real environmental samples are also provided in Fig. 8.

The peak current acquired for real samples was examined for chromium concentration (0–125 ppb) and summarized in Table 1. The concentrations of chromium from similar real samples were also measured using AAS. The obtained values were compared to those of the proposed technique in Table 1. In light of the high compatibility shown in Table 1, the proposed sensor with the CV technique can be reliably applied for direct redox sensing of hexavalent chromium.

**Table 1:** Chromium content in real spiked* samples.

S. No.	Real sample name	Cr(VI) content (ppb) (by developed sensor technique)	Cr(VI) content (ppb) (by AAS)
1	Real sample 1 (R.O water from GJUS&T, Hisar, India)	0	0
2	Real spiked sample 2 (Ghaggar Canal water, Hisar, India)	124.37	130
3	Real spiked sample 3 (Yamuna Canal water, Delhi, India)	127.06	133
4	Real spiked sample 4 (Sewage water, Hisar, India)	103.67	110

*Spiked with 100 ppb Cr(VI).

The prepared electrode was stable for two months while stored safely at proper conditions. Fabricated sensor and optimized technique have shown competent figure of merit as compared to other reported techniques and nanomaterials. Dominguez-Renedo *et al*. have detected hexavalent chromium using silver nanoparticles-modified carbon screen printed electrode. A detection limit of 40 ppb was achieved using differential pulse voltammetry technique^32^. In another research work, Cr (VI) was sensed up to 5 ppb using gold nanoparticle modified screen printed electrode with square wave voltammetry^33^. Jin and group have utilized amperometry method using fabricated gold nanoparticle decoratedtitania nanotube arrays for selective determination of hexavalent chromium in a concentration of 1.5 ppb^34^. Sari and co-workers have employed gold nanoparticles-graphene nanocomposites modified electrodes and impedance spectroscopy for electrochemical sensing of hexavalent chromium^35^. A limit of detection of 100 ppb was attained using gold nanoparticle-electrodeposited indium tin oxide (ITO) electrodes by Tsai *et al*. using cyclic voltammetry technique^36^. The fabricated nanochip sensor have shown a high sensitivity of 25.88 µA cm^−2^ ppb^−1^ (using CV technique) and a detection limit of 9.5 ppb with a linear range of 50–400 ppb. Based on the obtained results, the proposed sensor and technique can be effectively utilized for determining hexavalent chromium in real and laboratory samples.

## Materials and Methods

### Synthesis and characterization of manganese oxide nanoparticles

All chemicals were from Sigma Aldrich and used without further purification. The manganese oxide nanoparticles were synthesized using manganese sulphate (MnSO_4_) and hexamine (HMTA) by a refluxing method^10^. Solutions for each chemical were prepared separately as 0.1 M in 50 ml distilled water and mixed for 30 minutes under constant stirring at a solution pH of 10 (via adjustment with NaOH solution). The solution of a particular pH was then transferred to a round bottom flask connected to the condenser with a refluxing unit. The refluxing temperature was maintained at 80 °C for 5 h. The dark brown precipitates formed after completion of the reaction were cooled to room temperature. Their precipitates were collected from a beaker by removing the supernatant after being kept undisturbed overnight. These precipitates were filtered and washed with distilled water and ethanol three times to remove the impurities. The obtained manganese hydroxide nanoparticles were dried at 60 °C for 3 h. The dried nanoparticles were then placed in a furnace for calcination at 600 °C for 1 h in order to obtain manganese oxide nanoparticles. The synthesized manganese oxide nanoparticles were analysed for their structural, elemental, optical, topological, and morphological behaviour by FESEM, FTIR, particle size analysis, and XRD.

### Fabrication and evaluation of the Mn_3_O_4_/Nafion/Au electrode for redox sensing of hexavalent chromium

Synthesized manganese oxide nanoparticles were evaluated for their electro-catalytic activity against a redox change of hexavalent chromium. The bare gold electrode was coated with manganese oxide nanoparticles. Nafion (5 wt %) was applied as a binding agent to tightly hold the nanoparticles on the surface of the gold electrode. Slurry of manganese oxide nanoparticles in water was prepared and physically adsorbed to the surface of the gold electrode. This electrode was further dried under a heating lamp at an approximate temperature of 50 °C for the next 4 h. After drying, 1 µL of Nafion was applied to the surface of the Mn_3_O_4_/Au electrode for tight binding of nanoparticles on the surface of the Au electrode. This Mn_3_O_4_/Nafion/Au electrode was further dried for the next 2 h under the same temperature. The fabricated Mn_3_O_4_/Nafion/Au electrode was evaluated for its electro-catalytic activity in the redox change of the hexavalent chromium. A conventional three electrode system was used for characterization of the electrochemical properties. Platinum wire was applied as a counter electrode against Ag/AgCl (sat. KCl) as a reference electrode with a fabricated Mn_3_O_4_/Nafion/Au electrode as a working electrode. Autolab Potentiostat and Galvanostat with Nova software were employed for electrochemical experiments. Cyclic voltammetry (CV) is considered an efficient and robust tool for all electrochemical experiments.

A standard stock solution of hexavalent chromium was prepared using chromium trioxide. All solutions of hexavalent chromium were prepared in double distilled water and used as such for further evaluation. The effect of solution (containing analyte) pH on the CV signal was studied with the help of the fabricated Mn_3_O_4_/Nafion/Au electrode. All QA parameters (such as scan rate, analyte concentration, linear range, detection limit, sensitivity, and selectivity) were also standardized for the proposed technique. Cyclic voltammograms were collected at different scan rates and varying analyte concentration. The effect of the potential interferences was examined to assess the selectivity of the fabricated Mn_3_O_4_/Nafion/Au electrode towards redox sensing of hexavalent chromium. The CV was acquired for chromium only and in the presence of other possible interfering ions as well. Real environmental samples were also analysed (after adjusting the pH to 2) using a fabricated Mn_3_O_4_/Nafion/Au electrode with the proposed technique without any pre-treatment. The results were then compared against those obtained using other conventional techniques, such as AAS.

### Statistical parameters

All figures were designed in Origin Lab Professional V 2015 software. All electrochemical experiments were executed in triplicate. The obtained data were statistically analysed using Origin Lab Professional V 2015 software.

## Conclusion

Mn_3_O_4_ nanochips were synthesized using a refluxing technique and characterized for their elemental, morphological, topological, and structural properties. These characterized Mn_3_O_4_ nanochips were assessed for electrocatalytic activity through direct reduction of Cr(VI). Cyclic voltammetry is considered a novel, simple, and easy to operate analytical tool for direct determination of hexavalent chromium in real and environmental samples. Synthesized manganese oxide nanochips were coated on the surface of an Au electrode with the help of Nafion as a binding agent. The fabricated Mn_3_O_4_/Nafion/Au electrode was characterized electrochemically for analytical determination of Cr(VI). The fabricated sensor showed ultra-sensitivity of 25.88 µA cm^−2^ ppb^−1^ with a detection limit of 9.5 ppb. The linear range of 50–400 ppb was reproduced by fabricated the sensor with a relative standard deviation (RSD) less than 2 and a R^2^ value greater than 0.9. The fabricated sensor was established to be selective towards hexavalent chromium. Real spiked environmental samples were also analysed using the proposed technique and fabricated sensor, and results were in close agreement with conventional standard techniques. *For the first time*, a novel CV-based, direct determination technique for Cr(VI) was developed using Mn_3_O_4_ nanochips as an efficient electrocatalyst. Based on this ultrasensitive and selective determination method, the concentrations of hexavalent chromium were determined from both real and laboratory samples under various conditions.
